# A nationwide survey on the curriculum and educational resources related to the Clinical Skills Test of the Korean Medical Licensing Examination: a cross-sectional descriptive study

**DOI:** 10.3352/jeehp.2025.22.11

**Published:** 2025-03-13

**Authors:** Eun-Kyung Chung, Seok Hoon Kang, Do-Hoon Kim, MinJeong Kim, Ji-Hyun Seo, Keunmi Lee, Eui-Ryoung Han

**Affiliations:** 1 Department of Medical Education, Chonnam National University Medical School, Gwangju, Korea; 2 Department of Medical Education, Kangwon National University College of Medicine, Chuncheon, Korea; 3 Department of Family Medicine, Korea University Ansan Hospital, Ansan, Korea; 4 Department of Medical Education, Kosin University College of Medicine, Busan, Korea; 5 Department of Neurology, Kosin University College of Medicine, Busan, Korea; 6 Department of Pediatrics, Gyeongsang National University College of Medicine, Jinju, Korea; 7 Gyeongsang Institute of Health Sciences, Gyeongsang National University, Jinju, Korea; 8 Department of Family Medicine, Yeungnam University College of Medicine, Daegu, Korea; The Catholic University of Korea, Korea

**Keywords:** Clinical clerkship, Clinical competence, Cross-sectional studies, Medical licensure, Republic of Korea, Undergraduate medical education

## Abstract

**Purpose:**

The revised Clinical Skills Test (CST) of the Korean Medical Licensing Exam aims to provide a better assessment of physicians’ clinical competence and ability to interact with patients. This study examined the impact of the revised CST on medical education curricula and resources nationwide, while also identifying areas for improvement within the revised CST.

**Methods:**

This study surveyed faculty responsible for clinical clerkships at 40 medical schools throughout Korea to evaluate the status and changes in clinical skills education, assessment, and resources related to the CST. The researchers distributed the survey via email through regional consortia between December 7, 2023 and January 19, 2024.

**Results:**

Nearly all schools implemented preliminary student–patient encounters during core clinical rotations. Schools primarily conducted clinical skills assessments in the third and fourth years, with a simplified form introduced in the first and second years. Remedial education was conducted through various methods, including one-on-one feedback from faculty after the assessment. All schools established clinical skills centers and made ongoing improvements. Faculty members did not perceive the CST revisions as significantly altering clinical clerkship or skills assessments. They suggested several improvements, including assessing patient records to improve accuracy and increasing the objectivity of standardized patient assessments to ensure fairness.

**Conclusion:**

During the CST, students’ involvement in patient encounters and clinical skills education increased, improving the assessment and feedback processes for clinical skills within the curriculum. To enhance students’ clinical competencies and readiness, strengthening the validity and reliability of the CST is essential.

## Graphical abstract

[Fig f1-jeehp-22-11]

## Introduction

### Background/rationale

Assessments for licensure should reflect the competencies patients expect from physicians, be patient-centered, and consider the evolving nature of medical education [1]. In clinical practice, serious issues often arise from deficiencies in a physician’s communication skills and medical professionalism that do not reflect patients’ expectations. To strengthen the assessment of clinical competence and patient–physician interaction skills, the Korean Medical Licensing Examination (KMLE) revised its Clinical Skills Test (CST) in its 86th iteration, which was administered in 2021. The exam was redesigned to incorporate standardized patient encounters by replacing discrete technical skill items with essential technical skills integrated into some patient encounter sections [2].

Introducing new assessment systems often leads to curriculum changes. The CST of the KMLE, which was implemented in 2009, had a major impact on medical education in Korea [3], with many schools setting up clinical skills centers and adding clinical skills education, such as procedural skills training and standardized patient exams for third- and fourth-year students. Similarly, after introducing the 2004 clinical skills assessment in the United States, schools expanded assessments using standardized patients and increased financial investment in exam development and patient training [4]. China and Taiwan have also strengthened clinical training, increasing resources for standardized patient training and assessment facilities [5,6].

However, despite the transition to a patient-centered CST in 2021, little research has explored how medical education in Korea has evolved. Before the CST was revised, schools had already improved clinical skills assessments by improving the authenticity of standardized patient scenarios to promote patient-centered care or providing hybrid models to create more realistic clinical situations [7,8].

### Objectives

This study investigated potential curricular changes in response to the CST revision, focusing on its impact on medical education curricula and resources. Additionally, it aimed to identify specific aspects of the revised CST that medical schools sought to improve. It elicited insights from key representatives overseeing clinical clerkships, assessments, and training resources at medical schools to address the following research questions: (1) How has the CST revision, with its emphasis on patient-centered care, influenced clinical clerkships, clinical skills assessments, and training resources at medical schools? (2) How do medical schools perceive the need to improve the CST to support student education?

## Methods

### Ethics statement

The Institutional Review Board (IRB) of Chonnam National University Hospital (IRB no., CNUH-2023-422) approved this study. The researchers informed all participants about the study’s purpose and content and the participants provided consent before completing the survey.

### Study design

The researchers conducted a cross-sectional descriptive study to survey the curricula and educational resources related to the CST at 40 medical schools across Korea. Among the 40 medical schools that provide basic medical education, 38 function solely as a college of medicine, one operates exclusively as a graduate school of medicine, and the remaining institution offers both programs concurrently.

### Setting

The researchers asked each representative faculty member of the Regional Consortium for Standardized Patient Programs to distribute the survey to faculty responsible for clinical clerkship, skills education, or assessment at participating schools. We also sent follow-up reminder emails and text messages to encourage completion. The researchers collected responses between December 7, 2023 and January 19, 2024.

### Participants

The survey targeted faculty members responsible for clinical clerkships, skills education, or assessment at each medical school.

### Variables

The survey covered respondent demographics, institutional details, clinical clerkship, clinical skills assessments, clinical skills centers, and CST improvements. Questions on clinical clerkship explored preliminary student–patient encounters, the integration of CST items into curricula, and additional training for the objective structured clinical examination (OSCE) items not currently included in the CST. The items regarding simulated clinical skills assessments addressed exam timing, frequency, composition, administration, resources, assessors, grading, feedback, and retakes. For clinical skills centers, it examined establishment year, size, staffing, and usage. The survey also examined changes in training and assessments since the CST’s introduction in 2009, the patient-centered update in 2021, and its impact on skills centers. The survey invited participants to provide open-ended feedback on CST items and exam administration.

### Data sources/measurement

The research team reviewed the 2012 study, “The analysis on the impact of CST in KMLE and strategies for improvement,” conducted by the Research Institute for Healthcare Policy of the Korean Medical Association [9] and used it as a basis for developing the survey, focusing on the curriculum and educational resources related to the 2021 CST revision (Supplement 1). The survey included dichotomous (2-point) and polytomous (5-point) rating scales, multiple-choice questions, and open-ended questions. For analytical purposes, we reverse-coded the survey’s 5-point Likert scale, ranging from 1 (“strongly disagree”) to 5 (“strongly agree”). The Cronbach’s α for the 5-point Likert scale measuring the extent of changes in clinical clerkship, clinical performance examination (CPX), and OSCE was 0.699. Response data to survey is available at Dataset 1.

### Bias

Researchers minimized selection bias by targeting faculty members most relevant to the CST and working with lead professors at each school within the regional consortia.

### Statistical methods

Researchers performed statistical analysis using IBM SPSS ver. 26.0 (IBM Corp.) and summarized data using frequency analysis and descriptive statistics. For comparative analyses, the researchers used the paired t-test for changes in clinical clerkship and skills assessments following the introduction of the CST and patient-centered CST in 2009 and 2021, respectively.

## Results

### Participants

The study’s participants were the 40 faculty members responsible for clinical clerkships at all 40 medical schools nationwide, with a 100% response rate (Table 1). Most respondents (n=26, 65.0%) were directors of clinical education centers or clinical skills centers. Among these 26 directors, internal medicine was the most common specialty, representing 6 schools (23.1%), followed by emergency medicine, neurology, and surgery, each representing 3 schools (11.5%), and family medicine, obstetrics and gynecology, pediatrics, and medical education, each representing 2 schools (7.7%).

### Main results

#### Clinical clerkship

Table 2 shows that 38 schools (95.0%) reported conducting preliminary student–patient encounters in an average of 7.1 courses (range, 2–24 courses). The most common courses for these encounters included internal medicine, obstetrics and gynecology, family medicine, psychiatry, neurology, pediatrics, emergency medicine, and general surgery. Additionally, 14 schools (35.0%) operated standardized patient programs for educational purposes only, with each school managing an average of 24.1 standardized patients (range, 1–74). Of 40 schools, 39 (97.5%) included CPX items, while 36 (90.0%) included OSCE items.

Table 3 lists the OSCE items that were excluded from the CST but deemed essential since September 2021, indicating that Foley catheter insertion, electrocardiogram interpretation, and aseptic gowning and gloving are the most cited.

#### Simulated clinical skills assessment

All schools conducted simulated clinical skills assessments in the fourth year (40 schools, 100%), and almost all did so during the third year (38 schools, 95.0%). Thirteen schools (32.5%) conducted assessments in the second year, and 3 schools (7.5%) in the first year (Table 4). Exam formats varied: 16 schools (40.0%) combined 9 CPX and 1 OSCE item in the fourth year, while others used CPX-only, OSCE-only, or mixed formats.

In the fourth year, schools and the consortium jointly administered most assessments (56.8%). Schools mainly funded assessments through their budgets. Standardized patients primarily conducted CPX evaluations, while faculty assessed OSCEs. The most common evaluation method in the fourth year was incorporating assessment into subject credits (69.4%), followed by using them for subject credits or graduation qualification (16.7%) or for pass/fail (11.1%).

Faculty delivered feedback on assessment results mainly through one-on-one sessions with students, written feedback, class-wide feedback, or group discussions, with most schools using 1 or 2 methods, 5 using 3, and 1 employing all 4. Additionally, 38 schools (95.0%) offered remediation for low clinical skills scores, including video feedback, intensive training with standardized patients, counseling, guided learning, retests, or assignments, followed by further feedback (data not in Table 4).

#### Clinical skills centers

All 40 schools had separate clinical skills centers established between 2001 and 2018. Sixteen schools expanded or renovated their centers between 2008 and 2023. Three schools had 3 clinical skills centers, and 1 had 2. The average center occupied 767.5 m^2^, with sizes ranging from 83.3 to 2,826.4 m^2^. Centers averaged 1.5 full-time and 0.6 contract staff members. Thirty-eight schools reported using clinical skills centers for education. Most trained third- and fourth-year medical students, while 11 trained first-year students, 16 trained second-year students, and 1 school trained pre-medical first-year students (Table 5).

#### Perceptions of changes in the CST of the KMLE

The perceived impact of the CST on clinical clerkship significantly decreased from 4.03±0.90 in 2009 to 3.54±0.85 in 2021 (P=0.001). Similarly, the scores for CPX also decreased significantly (4.18±0.72 to 3.62±0.88, P=0.002), while that for OSCE exhibited a nonsignificant decrease (4.08±0.86 to 3.80±0.85, P=0.078) (Table 6).

#### Suggestions for improving the CST of the KMLE

Six schools (26.1%) suggested reintroducing the interstation written test or incorporating medical record assessments to improve the evaluation beyond the checklist-based system. They also recommended aligning CPX items with essential primary care tasks and prioritizing clinical competence for real-world practice over exam-specific performance.

Six schools (26.1%) proposed improving the reliability of the CST by increasing the difficulty and discrimination of exam items, strengthening the reliability and objectivity of standardized patient evaluations, and reassessing the appropriateness of the 12-minute exam duration to ensure adequate evaluation of clinical performance. Three schools (13.0%) suggested reinstating essential OSCE items and expanding the integration of OSCE and CPX items.

Regarding exam administration, 6 schools (35.3%) proposed securing additional exam sites and shortening the overall CST period. In addition, 5 schools (29.4%) suggested increasing the number of available exam opportunities and allowing students greater flexibility in choosing their exam dates (or times). Other suggestions highlighted the difficulty of securing standardized patients for CPX education, especially in rural areas. Additionally, respondents emphasized the need to adjust assessments of the patient–physician relationship to account for students with interpersonal difficulties. They also expressed concerns regarding the CST’s sustainability and effectiveness, given the anticipated increase in exam candidates due to expanding medical school enrollment.

## Discussion

### Key results

After the patient-centered CST was implemented as part of the KMLE in 2021, nearly all schools in this study implemented preliminary student–patient encounters into their clinical clerkship programs. Clinical skills assessments primarily occur during the third and fourth years of medical school. Post-exam, most schools offer remedial education. All schools have clinical skills centers, with ongoing improvements such as expansions, additional facilities, and increased staffing.

Faculty members overseeing clinical clerkships reported that the extent of changes in clinical education and skill assessment was small following the implementation of the patient-centered CST in 2021. Participants offered several suggestions to improve the CST, including reintroducing the interstation written test or implementing patient record assessments. Other recommendations focused on strengthening the assessments’ validity and reliability.

### Interpretation

The increase in preliminary student–patient encounters in core clinical subjects since the adoption of the patient-centered CST in the KMLE marks significant progress. Before the CST, clinical clerkships were largely observational, with such encounters and medical record documentation accounting for only 5% of training [10]. Studies indicate that the CST’s introduction has improved students’ communication skills and attitudes toward patients [3]. Despite the recent discontinuation of the US Step 2 Clinical Skills Assessment, proponents argue that clinical skills assessments remain essential for demonstrating competence and building patient trust [11]. These exams also encourage a greater focus on clinical education, enhancing training effectiveness and promoting learning [1,11].

Although schools predominantly administer clinical skills assessments during the fourth year before the national exam, some schools include simplified practical exams and training for first- and second-year students. Previous studies have reported a focus on third and fourth-year students, with practical exams in the first year at 1 school and in the second year at 7 schools [3]. While more schools now offer early-year exams, reducing OSCE items on the national exam may limit further expansion. However, integrated 6-year medical curricula could enable more balanced clinical skills education across all years, guided by faculty priorities for OSCE content.

Since the adoption of the patient-centered CST in the KMLE, all schools have provided feedback and remedial education through one-on-one sessions, group feedback, and post-exam reviews. Simulated clinical skills assessments aim to evaluate students’ clinical competence and offer feedback to ameliorate individual performance and improve educational curricula [12]. Notably, low communication scores in standardized patient assessments can increase the likelihood of patient complaints in clinical settings [13], emphasizing the need for early and frequent evaluations of communication and interpersonal skills during medical school. Faculty members report fewer changes in clinical skills education and assessment since the patient-centered CST’s implementation compared to the initial CST introduction, likely due to earlier integration of standardized patients, role-playing, and simulated clinical skills assessments before the CST’s introduction [3].

Participants suggested several improvements to increase the impact of the CST on school curricula. These include reinstating interstation written tests and patient record assessments to improve the evaluation of clinical competence, creating scenarios relevant to primary care, and focusing on practical skills required in actual clinical settings. To enhance reliability, faculty members suggested adjusting test difficulty, improving the objectivity of standardized patient evaluations, and conducting the CST across multiple test dates for consistency. Regular training and quality management for standardized patients are necessary to ensure realistic role performance. Reducing checklist items and adding written tests or patient record assessments could facilitate further assessments of critical knowledge and interpretation skills [14,15].

### Limitations

This study has several limitations. First, it relied on mail surveys, which led to 1 to 3 unanswered items for some questions. To improve clarity, researchers revised the survey 3 times during the initial research. Most unanswered items involved specific details about individual schools, making it challenging to provide detailed descriptions. Second, surveys alone cannot comprehensively analyze changes in the CST of the KMLE curricula across 40 medical schools. Future focus group interviews could offer deeper insights into these changes. Third, the study limited feedback to key representatives for clinical clerkships. Future studies should include students preparing for the CST and residents and faculty members involved in clinical practice education with CST experience. Lastly, it was difficult to compare faculty perceptions of the clinical education and assessment before and after the CST revision concerning institutional size (e.g., admission quotas) and management system (e.g., public versus private institutions). Comparing objective indicators of the curriculum and resources based on institutional size or management system could enhance the generalizability of the findings.

### Generalizability

This study makes a significant contribution by examining the current state of medical school curricula and changes prompted by the CST’s shift to a patient-centered format in the KMLE. By gathering input from key clinical clerkship representatives at 40 medical schools nationwide, it offers valuable insights into the evolution of medical education in response to the CST.

### Suggestions

As the CST has been in effect for 15 years, analyzing its outcomes and conducting ongoing research involving students, residents, and faculty is essential to ensure its continued development and effectiveness.

### Conclusion

The CST of the KMLE has significantly enhanced students’ opportunities for patient interactions and clinical skills education while promoting remedial education through clinical competence assessments and feedback. To ensure that the CST of the KMLE effectively evaluates medical graduates’ clinical performance and provides strong learning motivation for future clinical competence development, continuing efforts to strengthen its validity and reliability are essential.

## Figures and Tables

**Figure f1-jeehp-22-11:**
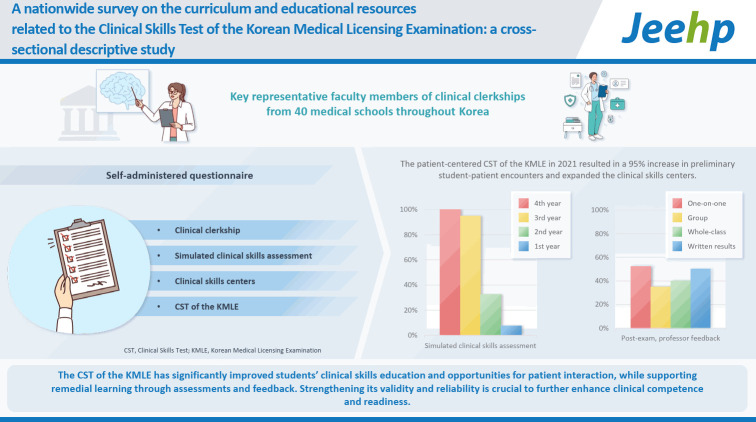


**Table 1.** Characteristics of key representatives of clinical clerkships at medical schools (N=40)

Characteristic	Value			
Gender				
Male	28 (70.0)			
Female	12 (30.0)			
Age (yr)	48.5±4.5			
Teaching experience (yr)	14.2±5.6			
Academic rank				
Professor	20 (50.0)			
Associate professor	17 (42.5)			
Assistant professor	3 (7.5)			
Administrative position^a)^				
Dean or associate dean	4 (10.0)			
Chair of medicine or chair of clinical medicine	3 (7.5)			
Director of education and training or director of education and research	2 (5.0)			
Director of the clinical education center or clinical skills center	26 (65.0)			
Director of clinical practice	2 (5.0)			
Other	6 (15.0)			

Values are presented as number (%) or mean±standard deviation.

a) These items allowed multiple responses.

**Table 2.** Clinical clerkship status in medical schools nationwide (N=40)

Item	Value			
Students’ preliminary encounters				
Yes	38 (95.0)			
No	2 (5.0)			
Subjects of students’ preliminary encounters				
Internal medicine	30 (78.9)			
Obstetrics and gynecology	18 (47.4)			
Family medicine	14 (36.8)			
Psychiatry	14 (36.8)			
Neurology	13 (34.2)			
Pediatrics	12 (31.6)			
Emergency medicine	12 (31.6)			
General surgery	10 (26.3)			
SP program for educational purposes only				
Yes	14 (35.0)			
No	26 (65.0)			
No. of SPs employed by the school	24.1±25.9 (1–74)			
Courses including CPX training^a)^				
Clinical practice by subject	36 (90.0)			
Introduction to clinical practice	28 (70.0)			
Other	4 (10.0)			
None	1 (2.5)			
Courses including OSCE training^a)^				
Clinical practice by subject	34 (85.0)			
Introduction to clinical practice	28 (70.0)			
Other	3 (7.5)			
None	3 (7.5)			

Values are presented as number (%) or mean±standard deviation (min–max). SP, standardized patient; CPX, clinical performance examination; OSCE, objective structured clinical examination.

a) These items allowed multiple responses.

**Table 3.** OSCE items recognized as necessary by the directors of clinical clerkships for education but not currently included on the CST of the KMLE (N=40)

OSCE item	No. (%)			
Foley catheter insertion	24 (60.0)			
ECG test	20 (50.0)			
Aseptic gowning and gloving	19 (47.5)			
Bone and joint splinting	15 (37.5)			
Chest X-ray presentation	14 (35.0)			
Emergency management of foreign body airway obstruction	13 (32.5)			
Lumbar puncture	11 (27.5)			
Injection (subcutaneous, intradermal, intramuscular)	9 (22.5)			
Endotracheal suction	7 (17.5)			
Incision and drainage of abscess	6 (15.0)			
Visual acuity test	2 (5.0)			

OSCE, objective structured clinical examination; CST, Clinical Skills Test; KMLE, Korean Medical Licensing Exam; ECG, electrocardiogram.

**Table 4.** Simulated clinical skills assessment by medical schools nationwide (N=40)

Items	No. (%)			
Exam phase				
First year	3 (7.5)			
Second year	13 (32.5)			
Third year	38 (95.0)			
Fourth year	40 (100.0)			
No. of exams^a)^				
1	2 (5.0)			
2	12 (30.0)			
3	20 (50.0)			
4	6 (15.0)			
No. of exams in the same format as the KMLE^a)^				
1	11 (27.5)			
2	3 (7.5)			
3	2 (5.0)			
Administration^a),b)^				
Medical school	1 (2.7)			
Regional consortium	15 (40.5)			
Both	21 (56.8)			
Funding^c)^				
Medical school budget	33 (82.5)			
External funds	7 (17.5)			
University budget	8 (20.0)			
CPX assessor^c)^				
Standardized patients	37 (92.5)			
Professors	15 (37.5)			
Both	12 (30.0)			
OSCE assessor				
Professors	37 (92.5)			
Standardized patients	2 (5.0)			
Both	1 (2.5)			
Evaluation method^a),b)^				
Subject credits only	25 (69.4)			
Pass/fail only	4 (11.1)			
Subject credits or qualification criteria for graduation	6 (16.7)			
Pass/fail or qualification criteria for graduation	1 (2.8)			
Professor feedback^c)^				
One-on-one (in-person)	21 (52.5)			
Group (in-person)	14 (35.0)			
Whole-class (in-person)	16 (40.0)			
Written	20 (50.0)			
No. of feedback methods				
1	16 (40.0)			
2	18 (45.0)			
3	5 (12.5)			
4	1 (2.5)			
Retest availability				
Yes	20 (50.0)			
No	20 (50.0)			

KMLE, Korean Medical Licensing Examination; CPX, clinical performance examination; OSCE, objective structured clinical examination.

a) This information corresponds to the fourth-year exam.

b) Missing values are excluded.

c) These items allowed multiple responses.

**Table 5.** Clinical skills centers at medical schools nationwide (N=40)

Items	Value
Year established^a)^	
Before 2010	22 (62.9)
2010–2015	11 (31.4)
After 2015	2 (5.7)
Expansion and renovation^a)^	
Before 2015	5 (31.3)
2015 and after	11 (68.8)
No. of centers	
1	36 (90.0)
2	1 (2.5)
3	3 (7.5)
Area (m^2^)	767.5±652.4
Dedicated staff	
Regular	1.5±2.0
Temporary	0.6±0.6
Clinical training usage by academic year^a)^	
Pre-medical first year	1 (2.6)
Medical first year	11 (28.9)
Medical second year	16 (42.1)
Medical third year	37 (97.4)
Medical fourth year	38 (100.0)

Values are presented as number (%) or mean±standard deviation.

a) Missing values are excluded.

**Table 6.** Changes in the curriculum owing to the CST of KMLE as perceived by directors of clinical clerkships

Item	Since the introduction of the CST in 2009	Since the changes to the CST with a focus on patient-centered care in 2021	P-value
Clinical clerkship	4.03±0.90	3.54±0.85	0.001
CPX	4.18±0.72	3.62±0.88	0.002
OSCE	4.08±0.86	3.80±0.85	0.078

Values are presented as mean±standard deviation. Five-point Likert scale anchored with 1=strongly disagree and 5=strongly agree. CST, Clinical Skills Test; KMLE, Korean Medical Licensing Examination; CPX, clinical performance examination; OSCE, objective structured clinical examination.

## Footnotes

**Data availability**
Data files are available from Harvard Dataverse: https://doi.org/10.7910/DVN/GMJIWR Dataset 1. Response data to survey.

## Supplementary materials

Supplementary files are available from Harvard Dataverse: https://doi.org/10.7910/DVN/GMJIWR

Supplement 1. Survey on the curriculum and educational resources related to the Clinical Skills Test of the Korean Medical Licensing Examination.

Supplement 2. Audio recording of the abstract.
