# Supplementary material for: A nationwide survey on the curriculum and educational resources related to the Clinical Skills Test of the Korean Medical Licensing Examination: a cross-sectional descriptive study
Source: J Educ Eval Health Prof. 2025 Mar 13;22:11. doi: 10.3352/jeehp.2025.22.11 (PMC12042100; doi:10.3352/jeehp.2025.22.11)
Supplement: Supplementary file 2 — Supplement 1. Survey on the curriculum and educational resources related to the Clinical Skills Test of the Korean Medical Licensing Examination. [file jeehp-22-11-suppl1.docx]

**Supplement 1.** Survey on the curriculum and educational resources related to the Clinical Skills Test of the Korean Medical Licensing Examination

**Survey on Medical School Faculty in Charge of Clinical Training Regarding the Curriculum and Educational Resources for the Clinical Skills Test of the Korean Medical Licensing Examination**

| **Ⅰ** |  | **Basic information** |
| --- | --- | --- |

This section contains general questions about your personal information and affiliated university.

1. Gender

① Male ② Female

2. Age: ( ) years

3. Current academic rank

① Assistant professor

② Associate professor

③ Professor

4. Years of teaching experience: ( )

5. Administrative position

① Dean or associate dean

② Chair of medicine or chair of clinical medicine

③ Director of education and training or director of education and research

④ Director of clinical education center or director of clinical skills center

⑤ Director of clinical practice (internal medicine)

⑥ Director of clinical practice (surgery)

⑦ Other (please specify: ____________________)

6. Medical specialty: (__________________)

| **Ⅱ** |  | **Simulated Clinical Skills Assessment** |
| --- | --- | --- |

7. This section concerns the Simulated Clinical Skills Assessment, which is a comprehensive mock exam similar to the Clinical Skills Test of the Korean Medical Licensing Examination. Please describe the number and timing of the assessments conducted in the 2023 academic year, the composition of exam items, the administrative body, and the evaluation method.

| **Academic year of examinees** | **Total number of exam sessions** | **Iteration of the exam** | **Exam timing (month)** | **Number of exam items** | **Administrative body** | | **Evaluation method** |
| --- | --- | --- | --- | --- | --- | --- | --- |
|  |  |  |  |  | **School**  **only** | **Collaboration with consortium** |  |
| 3 | ( ) rounds | 1^st^ | Month  ( ) | CPX ( ) items  OSCE ( ) items |  |  |  |
|  |  | 2^nd^ | Month  ( ) | CPX ( ) items  OSCE ( ) items |  |  |  |
|  |  | 3^rd^ | Month  ( ) | CPX ( ) items  OSCE ( ) items |  |  |  |
| 4 | ( ) rounds | 1^st^ | Month  ( ) | CPX ( ) items  OSCE ( ) items |  |  |  |
|  |  | 2^nd^ | Month  ( ) | CPX ( ) items  OSCE ( ) items |  |  |  |
|  |  | 3^rd^ | Month  ( ) | CPX ( ) items  OSCE ( ) items |  |  |  |
| Other  ( year) |  |  |  |  |  |  |  |
| Other  ( year) |  |  |  |  |  |  |  |

※ Note

Additional rows can be added if there are more than three sessions per academic year. If there are no such cases, leave the space blank. If exams are also conducted for years other than the 3rd and 4th, please describe them in the rows labeled “Other.”

The administrative body is the institution responsible for planning the exam items, including item development, question creation, standardized patient training, and exam operations.

The evaluation method should be described as follows: subject name (credits), pass/fail system, qualification criteria for graduation, etc.

CPX: clinical performance examination, OSCE: objective structured clinical examination.

8. Who assesses the CPX in the simulated clinical skills assessment? (Multiple responses are allowed)

① Standardized patients

② Professors

③ Other (please specify: )

9. Who assesses the OSCE in the simulated clinical skills assessment?

① Standardized patients

② Professors

③ Other (please specify: )

10. From which source is the funding obtained for the simulated clinical skills assessment? (Multiple responses are allowed)

① Medical school budget

② External funds for medical school development

③ University budget

④ Other (please specify: )

11. How is feedback provided for the clinical skills assessment? (Multiple responses are allowed)

① One-on-one feedback from professors (in-person)

② Group feedback from professors (in-person)

③ One professor provides feedback to the whole class of students (in-person)

④ Feedback is provided in written results

⑤ Other (please specify: )

12. Is a retake available for the simulated clinical skills assessment?

① Yes

② No

13. Is passing the simulated clinical skills assessment required for graduation?

① Yes

② No

14. Please describe the educational methods your school currently implements for students with low scores in the simulated clinical skills assessment.

|  |
| --- |

15. Has the CPX of the simulated clinical skills assessment at your school changed in any way since the Clinical Skills Test of the Korean Medical Licensing Examination was introduced in 2009?

① Strongly agree

② Agree

③ Neutral

④ Disagree

⑤ Strongly disagree

15-1. If you have anything to add regarding the above item, please provide additional detailed comments below.

|  |
| --- |

16. Has the OSCE of the simulated clinical skills assessment at your school changed in any way since the Clinical Skills Test of the Korean Medical Licensing Examination was introduced in 2009?

① Strongly agree

② Agree

③ Neutral

④ Disagree

⑤ Strongly disagree

16-1. If you have anything to add regarding the above item, please provide additional detailed comments below.

|  |
| --- |

17. Has the CPX of the simulated clinical skills assessment at your school changed in any way since the patient-centered Clinical Skills Test of the Korean Medical Licensing Examination was adopted in 2021?

① Strongly agree

② Agree

③ Neutral

④ Disagree

⑤ Strongly disagree

17-1. If you have anything to add regarding the above item, please provide additional detailed comments below.

|  |
| --- |

18. Has the OSCE of the simulated clinical skills assessment at your school changed in any way since the patient-centered Clinical Skills Test of the Korean Medical Licensing Examination was adopted in 2021?

① Strongly agree

② Agree

③ Neutral

④ Disagree

⑤ Strongly disagree

18-1. If you have anything to add regarding the above item, please provide additional detailed comments below.

|  |
| --- |

19. Does your school independently manage standardized patients, regardless of consortium collaboration?

① Yes ☞ Proceed to question 19-1

② No ☞ Proceed to question 20

19-1. How many standardized patients are currently managed independently by your school (as of December 2023)?

( ) individuals

19-2. Is the standardized patient program also used for educational purposes (e.g., communication skills training using the “time in, time out” method, physical examination training using standardized patients, etc.)?

① Yes

② No

| **Ⅲ** |  | **Clinical clerkship** |
| --- | --- | --- |

The following questions are about clinical clerkship.

20. Are there any clinical clerkship courses in which students conduct the preliminary student–patient encounter?

① Yes ☞ Proceed to question 20-1

② No ☞ Proceed to question 21

20-1. How many clinical clerkship courses involve the preliminary student–patient encounter?

( ) courses

20-2. Please list the clinical clerkship courses that involve the preliminary student–patient encounter.

|  |
| --- |

21. Are the CPX components of the Clinical Skills Test of the Korean Medical Licensing Examination incorporated into the clinical clerkship curriculum?

① Yes ☞ Proceed to question 21-1

② No ☞ Proceed to question 22

21-1. In which courses are CPX components incorporated? (Multiple answers are allowed)

① Introduction to clinical practice

② Clinical practice by subject

③ Other (please specify: )

22. Are the OSCE components of the Clinical Skills Test of the Korean Medical Licensing Examination incorporated into the clinical clerkship curriculum?

① Yes ☞ Proceed to question 22-1

② No ☞ Proceed to question 23

22-1. In which courses are OSCE components incorporated? (Multiple answers are allowed)

① Introduction to clinical practice

② Clinical practice by subject

③ Other (please specify: )

23. Are there any OSCE items not currently included in the OSCE section of the Clinical Skills Test of the Korean Medical Licensing Examination (since September 2021) that you believe should be included in the curriculum for educational purposes?

① Yes ☞ Proceed to question 23-1

② No ☞ Proceed to question 24

23-1. Please select the items you think should be included in the curriculum (multiple answers are allowed).

| **OSCE item** | **Selection** | **OSCE item** | **Selection** |
| --- | --- | --- | --- |
| Visual acuity test |  | Incision and drainage of abscess |  |
| ECG test |  | Aseptic gowning and gloving |  |
| Lumbar puncture |  | Chest X-ray presentation |  |
| Bone and joint splinting |  | Emergency management of foreign body airway obstruction |  |
| Foley catheter insertion |  | Injection (subcutaneous, intradermal, intramuscular) |  |
| Endotracheal suction |  | Others ( ) |  |
| Others ( ) |  | Others ( ) |  |

24. Is there focused training on the CPX or OSCE for the Clinical Skills Test of the Korean Medical Licensing Examination offered in the 3rd or 4th year as an extracurricular program?

① Yes ☞ Proceed to question 24-1

② No ☞ Proceed to question 25

24-1. How is the timing and total duration of the training structured?

| **Academic year** | **Program** | **Total training time**  **(per student)** |
| --- | --- | --- |
|  |  |  |
|  |  |  |
|  |  |  |

25. Has the clinical clerkship at your school changed in any way since the Clinical Skills Test of the Korean Medical Licensing Examination was introduced in 2009?

① Strongly agree

② Agree

③ Neutral

④ Disagree

⑤ Strongly disagree

25-1. If you have anything to add regarding the above item, please provide additional detailed comments below.

|  |
| --- |

26. Has the clinical clerkship at your school changed in any way since the patient-centered Clinical Skills Test of the Korean Medical Licensing Examination was adopted in 2021?

① Strongly agree

② Agree

③ Neutral

④ Disagree

⑤ Strongly disagree

26-1. If you have anything to add regarding the above item, please provide additional detailed comments below.

|  |
| --- |

| **Ⅳ** |  | **Clinical skills center** |
| --- | --- | --- |

The following questions are about the clinical skills center at your school (or hospital).

27. Does your school have a separate clinical skills center?

① Yes ☞ Proceed to question 27-1

② No ☞ Proceed to question 31

27-1. Please specify the year of establishment and the area of the independent clinical skills center.

| **Name of the clinical skills center** | **Location (campus)** | **Year of establishment** | **Year of expansion or renovation** | **Area (size)** |
| --- | --- | --- | --- | --- |
|  |  |  |  | ( ) m^2^ |
|  |  |  |  | ( ) m^2^ |
|  |  |  |  | ( ) m^2^ |

28. Has the introduction of the Clinical Skills Test of the Korean Medical Licensing Examination significantly influenced the establishment or expansion of the Clinical Skills Center?

① Strongly agree

② Agree

③ Neutral

④ Disagree

⑤ Strongly disagree

29. What is the composition of the official staff managing the clinical skills center?

Full-time regular staff: ( ) persons

Full-time contract staff: ( ) persons

Other (please specify):

30. Please specify the specific time and programs during which the clinical skills training programs are incorporated into your school’s curriculum through the clinical skills center.

| **Academic year** | **Program** |
| --- | --- |
|  |  |
|  |  |
|  |  |
|  |  |

| **Ⅴ** |  | **Suggestions for improving the Clinical Skills Test of the Korean Medical Licensing Examination** |
| --- | --- | --- |

31. If there are any improvements to be made to the items in the current Clinical Skills Test of the Korean Medical Licensing Examination, please provide specific details.

|  |
| --- |

32. If there are any improvements to be made regarding the operation of the current Clinical Skills Test (e.g., exam schedule, exam duration, exam method), please provide specific details.

|  |
| --- |

33. If you have any additional comments or suggestions regarding the current Clinical Skills Test, please feel free to provide them below.

|  |
| --- |

| **Ⅵ** |  | **Institutional information** |
| --- | --- | --- |

34. Please provide the name of your school.

|  |
| --- |

35. Please provide the 2023 admission quota for your school.

|  |
| --- |

Thank you very much for your participation in this survey.
